# Integrated Transcriptomics and Proteomics Reveal Amino Acid Composition and Protein Expression Profiles of *Crassostrea hongkongensis* and *Crassostrea ariakensis*

**DOI:** 10.3390/foods15203606

**Published:** 2026-10-10

**Authors:** Shaojuan Liu, Huifang Ma, Ling Lin, Shaodong Zeng, Saihao Ren, Donghong Zhu, Matej Orešič, Xiaowei Pan, Jianzhi Ye

**Affiliations:** 1 Laboratory of Quality & Safety Risk Assessment on Agro-Products Processing (Zhanjiang), Ministry of Agriculture and Rural Affairs, Agricultural Products Processing Research Institute, Chinese Academy of Tropical Agricultural Sciences, Zhanjiang 524001, China; liushaojuan2023@163.com (S.L.); wolovexb@catas.cn (H.M.); linling408@163.com (L.L.); shaodongzeng@163.com (S.Z.); rshkyzy@foxmail.com (S.R.); 18900835518@163.com (D.Z.); 2 School of Medical Sciences, Faculty of Medicine and Health, Örebro University, SE-701 82 Örebro, Sweden; matej.oresic@oru.se; 3 Turku Centre for Biotechnology, University of Turku, FI-20520 Turku, Finland; 4 Turku Centre for Biotechnology, Abo Akademi University, FI-20520 Turku, Finland; 5 Department of Life Technologies, University of Turku, FI-20014 Turku, Finland

**Keywords:** oysters, amino acid, protein profiles, transcriptomics, proteomics, multi-omics integration

## Abstract

*Crassostrea hongkongensis* and *Crassostrea ariakensis* are commercially and nutritionally important oysters. However, species-specific differences in amino acid composition and protein expression profiles, and underlying mechanisms, remain poorly understood. Here, we integrated high-performance liquid chromatography (HPLC), transcriptomic, and proteomic analysis to address these objectives. *C. ariakensis* exhibited significantly higher total hydrolyzed amino acid contents than *C. hongkongensis,* with glycine, alanine, glutamic acid, proline and serine being the predominant amino acids in both species. Proteomic analysis identified 1371 differentially expressed proteins (DEPs) that were primarily enriched in proteasome, spliceosome, ribosome, and amino acid metabolism. Transcriptomic analysis screened 16,747 differentially expressed genes (DEGs), which were significantly enriched in the Notch signaling pathway, Ubiquitin mediated proteolysis, Neuroactive ligand-receptor interaction and Phagosome pathways. The integration of proteomics and transcriptomics jointly identified five common KEGG pathways, among which gene set enrichment analysis (GSEA) showed ECM–receptor interaction and folate biosynthesis to be significantly downregulated in *C. hongkongensis*, suggesting their potential involvement in shaping amino acid composition and protein abundance. Collectively, these findings provide a foundation for the further investigation of species-specific nutritional differences and their potential implications for oyster aquaculture.

## 1. Introduction

Oysters are among the most economically valuable and widely farmed bivalves worldwide and play an important role in fisheries and aquaculture. They are rich in nutrients such as amino acids, proteins, polysaccharides, and vitamins, making them highly popular among consumers [1]. In China, *C. hongkongensis* and *C. ariakensis* are closely related species within the genus *Crassostrea* and are widely cultivated in southern coastal and estuarine areas. Despite their morphological similarity, they exhibit obvious differences in habitat preference, growth rate, and stress tolerance [2,3,4]. *C. ariakensis* typically inhabits low-salinity, turbid estuarine environments, while *C. hongkongensis* is more adapted to relatively high-salinity coastal waters and grows faster. These ecological and physiological distinctions suggest underlying molecular and metabolic differences that may influence their nutritional composition.

The nutritional value of oyster meat mainly depends on its protein content and amino acid composition. Amino acids are the basic building blocks of proteins and can directly affect the taste characteristics of seafood. For example, free amino acids such as glutamate, glycine, alanine, and arginine are the main components determining the umami and sweet flavors that define oyster palatability [5]. However, it should be noted that the present study measured total hydrolyzed amino acids, which include both protein-bound and free amino acids. Recent studies have revealed significant interspecies differences in amino acid composition and in responses to environmental factors. In Pacific oysters (*Crassostrea gigas*), the main free amino acids (FAA) include taurine, glutamic acid, glycine, alanine, proline, and arginine, with glycine and glutamic acid being the most abundant protein-bound amino acids [6]. It is notable that taurine is the most abundant free amino acid, followed by glutamic acid, glycine, and alanine. A systematic comparison of *C. sikamea*, *C. hongkongensis*, and *C. ariakensis* showed that overall quality ranked *C. sikamea* > *C. hongkongensis* > *C. ariakensis*, indicating substantial variation in amino acid composition and protein expression even among closely related species [7]. Environmental factors also modulate amino acid content [8]. In *C. hongkongensis*, free amino acids serve as major intracellular osmolytes during salinity adaptation: total free amino acid content decreases in the gills and muscle but increases in the hemolymph under low salinity, while it increases in all tissues under high salinity. Taurine responds most strongly to low salinity, whereas alanine and glycine are key osmolytes for high-salinity adaptation [3]. Taurine contributes the most significantly to the response to low salinity, while alanine and glycine are the key osmotic regulatory substances for adapting to high salinity. A recent comparative study of four species of scallop bivalves further determined that glutamic acid, aspartic acid, alanine, and glycine are the key amino acids in response to high salinity stress [9]. These studies have emphasized the close relationship between amino acid composition and environmental adaptability. However, the species-specific differences in amino acid composition and protein expression profiles between *C. hongkongensis* and *C. ariakensis*, as well as the underlying molecular mechanisms under comparable environmental conditions, remain largely unknown.

In recent years, multi-omics techniques such as transcriptomics and proteomics have been widely employed to investigate the molecular mechanisms underlying the adaptability and nutritional quality regulation in bivalves. Tan et al. used transcriptomics and metabolome analyses to elucidate the molecular basis of growth regulation in selectively bred *Crassostrea gigas* strains [10]. The results have indicated that enhanced protein synthesis, fatty acid metabolism, and cell proliferation were key drivers of the rapid growth phenotype. Zhang et al. combined transcriptomic and metabolomic analysis and revealed that *C. hongkongensis* mainly adapts to high salinity through osmoregulation [2]. The differentially expressed genes (DEGs) were mainly enriched in NOD-like receptor signaling, NF-κB signaling, apoptosis, and amino acid metabolism pathways, and the non-specific immune enzyme activity was activated in response to high salinity stress. In the eastern oyster *Crassostrea virginica*, transcriptomics, proteomics, and physiological analyses revealed that ocean acidification can impair immune function and increase susceptibility to pathogen-induced mortality [11]. Multi-omics integration techniques have been widely applied to reveal the molecular basis of flavor and nutritional components. Chen et al. used 4D-FastDIA-based quantitative proteomics to identify protein biomarkers related to flavor characteristics in triploid and diploid oysters (*Crassostrea angulata*) [12]. Collectively, these studies demonstrate that multi-omics approaches provide a powerful means to explore the complex molecular mechanisms related to growth, stress resistance, and nutritional composition in oysters. Previous studies have separately reported species-specific nutritional traits or salinity-related molecular responses in oysters. However, no study has yet integrated HPLC-based amino acid profiling with transcriptomic and proteomic analyses to compare *C. hongkongensis* and *C. ariakensis* under identical environmental conditions. This integrated approach allows us to link amino acid composition directly to the underlying molecular regulatory mechanisms, providing a more comprehensive understanding of species-specific nutritional differences than single-omics or single-trait studies.

The specific research gap addressed in this study is the lack of integrated molecular evidence explaining why these two closely related oyster species differ in amino acid composition and protein expression profiles under comparable environmental conditions. We hypothesized that (i) *C. ariakensis* and *C. hongkongensis* would exhibit distinct amino acid profiles, particularly in flavor-related amino acids; (ii) these differences would be accompanied by species-specific expression patterns of genes and proteins involved in protein synthesis, degradation, and metabolism; and (iii) integrated transcriptomic and proteomic analyses would reveal key pathways linking molecular regulation to nutritional traits. In this study, we compared the total hydrolyzed amino acid compositions of *C. hongkongensis* and *C. ariakensis* using HPLC. Transcriptomics and proteomics were employed to elucidate the differences in protein abundance and transcriptional expression levels between the two species. Furthermore, we integrated the transcriptomic and proteomic results to reveal the molecular regulatory mechanisms underlying protein synthesis and metabolism. This study provides a clearer understanding of the molecular basis of species-specific differences in amino acid composition and protein expression profiles, offering a foundation for the further investigation of nutritional differences and their potential implications for oyster aquaculture.

## 2. Materials and Methods

### 2.1. Materials and Sample Collection

Fresh oyster samples of *Crassostrea hongkongensis* and *Crassostrea ariakensis* were collected from the Beibu Gulf (Zhanjiang, Guangdong Province, China; 21°41′ N, 108°48′ E) in August 2025. A total of six individuals per species were sampled. Environmental parameters at the collection site were: salinity 12%, temperature 31 °C, dissolved oxygen 5.28 mg/L, and pH 7.9. Following harvest, soft tissues were carefully excised from the shells, flash-frozen in liquid nitrogen, and stored at −80 °C until further analysis. For all analyses, three biological replicates per species were used. Each biological replicate consisted of tissue from a single individual and no pooling of samples was performed.

### 2.2. Determination of Amino Acids by HPLC

Twenty milligrams of lyophilized oyster sample were weighed and transferred quantitatively into a sealed hydrolysis tube. Then, 10.0 mL of 6 mol/L hydrochloric acid (analytical grade, prepared as a 1:1 *v*/*v* aqueous solution) was added, and the headspace was purged with high-purity nitrogen gas for 30 s to remove residual oxygen. Hydrolysis was performed at 110 °C for 22–24 h. After hydrolysis, the hydrolysate was cooled to room temperature, filtered through a 0.45 μm membrane into a 50 mL volumetric flask, and diluted to the mark with ultrapure water. An aliquot of 2.0 mL was transferred into a 50 mm-diameter glass weighing dish and evaporated under gentle heating at 85 °C until only a minimal residue remained. The residue was fully dissolved in 1.0 mL sodium citrate buffer and filtered through a 0.22 μm strainer. The final solution was used for amino acid determination using HPLC (Biochrom 30+, Biochrom Ltd., Cambridge, UK). This acid hydrolysis procedure measures total hydrolyzed amino acids, including both protein-bound and free amino acids; cysteine is largely destroyed during acid hydrolysis.

The HPLC system was equipped with a sodium-type cation exchange column (200 mm × 4.6 mm, 8 μm particle size). Separation was achieved using a stepwise elution program with three citrate buffers: buffer B1 (pH 3.20, 0.20 M), buffer B2 (pH 4.25), and buffer B5 (pH 6.45, 0.20 M), followed by a regeneration solution (0.04 M NaOH). The buffer flow rate was 25 mL/h, and the ninhydrin reagent flow rate was 10 mL/h. The column temperature was programmed at 55–65–77 °C, and the reaction coil temperature was 138 °C (ninhydrin derivatization temperature: 145 °C). Detection was performed at 570 nm and 440 nm using a UV-Vis detector (Biochrom 30+, Biochrom Ltd.) after post-column derivatization with ninhydrin. The injection volume was 20 μL.

For calibration, an amino acid standard solution (AAS18, Sigma-Aldrich, St. Louis, MO, USA) was diluted 5-fold with sodium citrate buffer, yielding final concentrations of 0.5 μM/mL for each amino acid (except cystine at 0.25 μM/mL). The standard mixture contained aspartic acid, threonine, serine, glutamic acid, glycine, alanine, cystine, valine, methionine, isoleucine, leucine, tyrosine, phenylalanine, histidine, lysine, arginine, and proline. Quality control was assessed by determining the limit of detection (3 pmol for aspartic acid at S/N = 2), retention time repeatability (CV 0.3% for arginine), and peak area repeatability (CV 1.0% for glycine and histidine). Three biological replicates were performed.

### 2.3. RNA Extraction

Total RNA of oyster samples was extracted and purified using Trizol reagent (Thermo Fisher, 15596018, Waltham, MA, USA) following the manufacturer’s procedure. The quantity and purity of total RNA were analyzed using Bioanalyzer 2100 and RNA 6000 Nano LabChip Kit (Agilent, Santa Clara, CA, USA, 5067-1511), and high-quality RNA samples with RIN number > 7.0 were used to construct sequencing library.

### 2.4. Transcriptome Sequencing

After total RNA was extracted, mRNA was purified from total RNA (5 μg) using Dynabeads Oligo (dT) (Thermo Fisher, San Jose, CA, USA) with two rounds of purification. Following purification, mRNA was fragmented and reverse-transcribed, and U-labeled second-stranded DNAs were synthesized. An A-base was added to the blunt ends of each strand, preparing them for ligation to the indexed adapters. Each adapter contained a T-base overhang for ligating the adapter to the A-tailed fragmented DNA. Dual-index adapters were ligated to the fragments, and size selection was performed with AMPureXP beads. After the PCR amplification of cDNA, the average insert size of the final cDNA libraries was 300 ± 50 bp. Finally, 2 × 150 bp paired-end sequencing (PE150) was performed on an Illumina Novaseq^TM^ 6000 (Illumina, Inc., San Diego, CA, USA) following the vendor’s recommended protocol.

Raw reads were filtered, trimmed, and quality-checked using Cutadapt (1.9). Sequence quality was assessed using FastQC (v 0.10.1), and the error rate, Q20, Q30, and GC content of the cleaned data were calculated. The mean Q30 percentage was 95.84%, and the GC content was 39.81%. Cleaned reads that passed quality control were used in downstream analyses. De novo assembly of the transcriptome was performed with Trinity (2.15). The quality of assembly was evaluated by BUSCO (5.4.4). All assembled unigenes were aligned against the non-redundant (Nr) protein (http://www.ncbi.nlm.nih.gov/), Gene ontology (GO) (http://www.geneontology.org), SwissProt (http://www.expasy.ch/sprot/, accessed on 10 March 2026), Kyoto Encyclopedia of Genes and Genomes (KEGG) (http://www.genome.jp/kegg/, accessed on 20 March 2026) and eggNOG (http://eggnogdb.embl.de/, accessed on 25 March 2026) databases using DIAMOND (2.0.15) with a threshold of Evalue < 0.00001. Unigene expression levels were quantified as TPM (Transcripts Per Kilobase of exon model per Million mapped reads). Cleaned reads were mapped back to the de novo assembled transcriptome using Salmon (1.9.0), with a mapping rate of 98.32%. Unigene differential expression analysis was performed using edgeR (3.40.2) with three biological replicates per species. Unigenes with a false discovery rate (FDR) < 0.05 and fold change > 2 or <0.5 were considered DEGs. GO function or KEGG pathway significant enrichment analyses were performed by mapping all significant DEGs to each GO term or KEGG pathway, and a hypergeometric test was applied to identify significantly enriched terms compared to the whole-genome background. Three biological replicates were performed. Each biological replicate was derived from an individual oyster.

### 2.5. Protein Extraction

Oyster samples were weighed and transferred into a 2 mL centrifuge tube. Steel beads, lysis buffer containing 8 M urea/50 mM Tris-HCl, and 1× Roche Cocktail were added. After the samples were placed on ice for 5 min, grinding and lysis were performed using a grinding instrument at 60 Hz for 2 min, followed by centrifugation at 20,000× *g* at 4 °C for 15 min. The supernatant was retained, and 10 mM DTT was added, followed by incubation in a water bath at 37 °C for 1 h. Then, 20 mM IAA was added, and the samples were placed in the dark for 30 min. Protein quality was assessed using the Bradford method. Trypsin was added at a protein-to-trypsin ratio of 50:1, and samples were incubated for 14–16 h at 37 °C. The enzymatically digested peptides were desalted using Waters solid-phase extraction cartridges and vacuum-dried. The dried peptide fractions were redissolved in pure water and stored at −20 °C for proteomic analysis.

### 2.6. Proteomic Analysis

The peptide mixture was diluted with solvent A (5% ACN, pH 9.8), injected, and separated using a Thermo Scientific UltiMate 3000 Binary Rapid Separation System (Thermo Fisher Scientific, San Jose, CA, USA). Gradient elution was performed at 0.3 mL/min: 5% to 21% solvent B (97% ACN, pH 9.8) for 38 min, 21.5% to 40% solvent B for 20 min, 40% to 90% solvent B for 2 min, 90% solvent B for 3 min, and 5% solvent B for 10 min equilibration. The elution peaks were monitored at 214 nm and fractions were collected every minute. Fractions were combined according to chromatograms, and ten fractions were obtained and freeze-dried. Then, the dried peptide samples were reconstituted with 0.1% formic acid and centrifuged at 20,000× *g* for 10 min. Separation was performed using a Thermo Scientific Vanquish Neo UHPLC system (Dionex Softron GmbH, Part of Thermo Fisher Scientific, Germering, Germany). The liquid-phase separated peptides were transferred to a Thermo Astral mass spectrometer (Thermo Fisher Scientific, Bremen, Germany) for acquisition in DIA (Data Independent Acquisition) mode. DIA-NN (version 1.9) was used to analyze the DIA data with a library-free method. MS/MS data were searched against protein sequences downloaded from the UniProt database (UniProt *Crassostrea gigas* reference proteome, release 2024_10). The *Crassostrea gigas* reference proteome (UniProt Proteome ID: UP000005408) was used as the search database because it is the most comprehensively annotated reference proteome available for the genus *Crassostrea*. Although *C. hongkongensis* and *C. ariakensis* are closely related to *C. gigas*, sequence divergence may reduce peptide identifiability and introduce potential quantitative bias. Therefore, the proteomic results should be interpreted with this limitation in mind. The protein identification credibility strategy was “Target–decoy” with a 1% FDR and only protein groups passing the filter criteria were used in downstream analysis.

Statistical analysis of proteomic data was performed using R software (version 4.0). Cluster heatmaps were drawn using the pheatmap package. PCA analysis was completed using the metaX package. Correlation analysis was conducted using the Pearson correlation coefficient with the cor package. For differential expression analysis, the study design consisted of two groups (*C. hongkongensis* and *C. ariakensis*) with three biological replicates per group. Therefore, Student’s *t*-test was used to compare protein abundances between the two groups. For proteomic comparisons, normality was assessed using the Shapiro–Wilk test and homogeneity of variance was assessed using Levene’s test. When both assumptions were met, Student’s *t*-test was used; otherwise, the Mann–Whitney U test was applied. All *p*-values were adjusted using the Benjamini–Hochberg (BH) method to control the false discovery rate. Proteins with adjusted *p* < 0.05 and |log2FC| ≥ 0.263 (FC ≥ 1.2 or ≤0.83) were considered differentially expressed proteins (DEPs). Based on the hypergeometric test, differences in enrichment of Gene Ontology (GO), KEGG Pathway, Reactome Pathway, and InterPro domains were analyzed. GSEA (v4.1.0) and MSigDB were used for gene set enrichment analysis with criteria of |NES| > 1, NOM *p*-value < 0.05, and FDR *q*-value < 0.25. Three biological replicates were performed. Each biological replicate was derived from an individual oyster.

### 2.7. Transcriptome and Proteomic Association Analysis

To identify shared KEGG pathways between transcriptomics and proteomics, a pathway-level overlap analysis was conducted on the KEGG pathways significantly enriched in both omics layers. A heatmap was used to present the detailed information of DEGs and DEPs within the shared pathways using R software (version 4.0).

### 2.8. Statistical Analysis

Data were analyzed using GraphPad Prism 10.0 and are presented as means ± standard error of mean (SEM) from three biological replicates. Student’s *t*-test was performed to evaluate significant differences between the two groups, with significance set at *p* < 0.05. For pairwise comparisons between the two species, normality was assessed using the Shapiro–Wilk test and homogeneity of variance using Levene’s test. Student’s *t*-test was used when assumptions were met; otherwise, the Mann–Whitney U test was applied. For multiple amino acid comparisons, *p*-values were adjusted using the Benjamini–Hochberg method. Significance was set at adjusted *p* < 0.05.

## 3. Results and Discussion

### 3.1. Compositions of Amino Acids in C. hongkongensis and C. ariakensis

**Figure 1:**
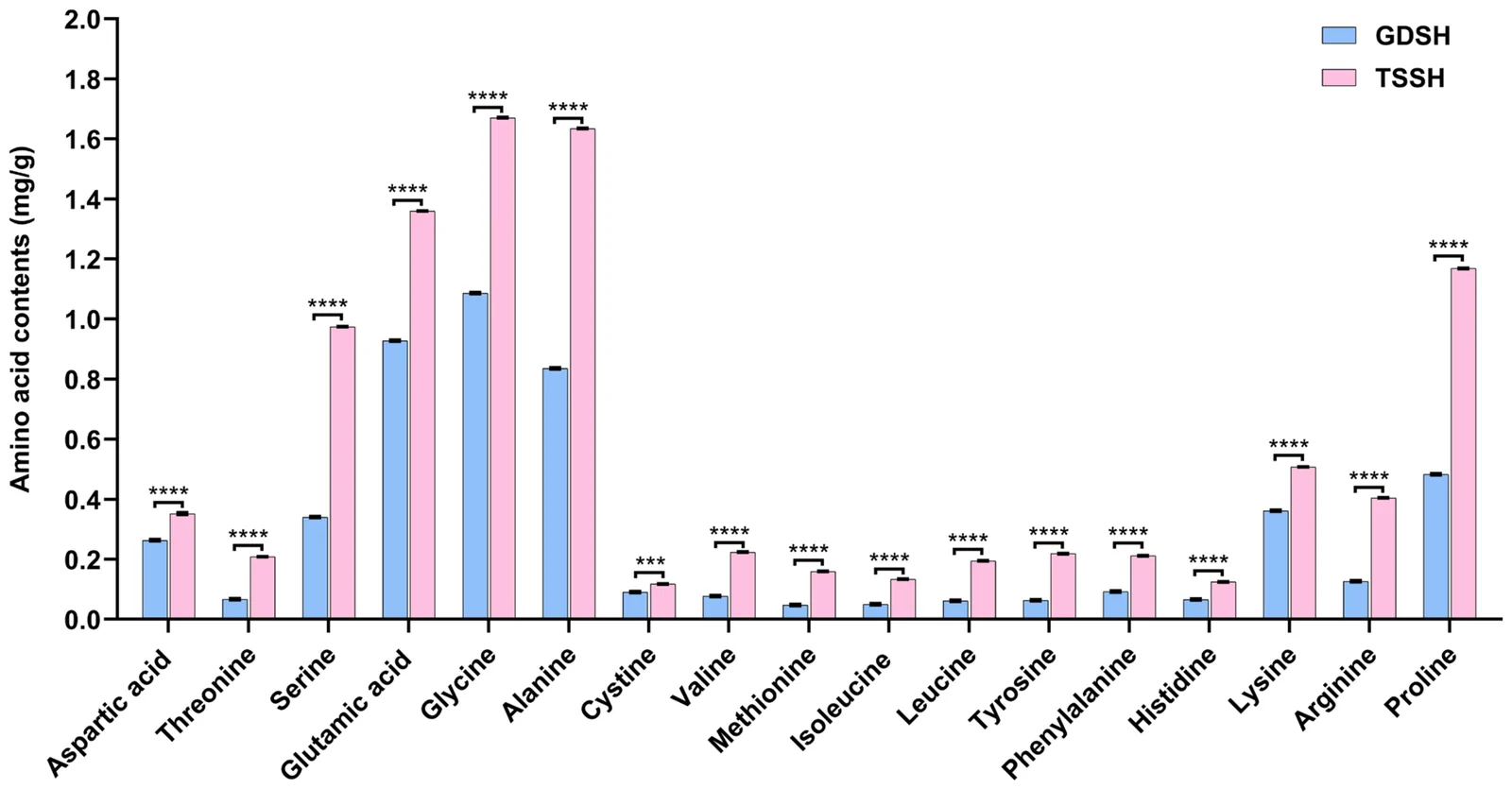
Compositions of amino acids in *C. hongkongensis* and *C. ariakensis*. GDSH: *C. hongkongensis*, TSSH: *C. ariakensis*. The significance of results was determined using Student’s *t*-test (*n* = 3). *** *p* < 0.001, and **** *p* < 0.0001 represent significant differences.

Amino acids are the basic units of proteins and can directly participate in protein synthesis. Oysters are valuable source of high-quality animal protein and are rich in essential amino acids [13]. From a food science perspective, the species-specific differences in amino acid composition and protein expression profiles may have direct implications for the nutritional quality and functional properties of oyster products. In this study, the amino acid compositions of the two species are shown in Figure 1. The total hydrolyzed amino acid content of *C. ariakensis* was significantly higher than that of *C. hongkongensis*. Specifically, the content of each measured amino acid was significantly higher in *C. ariakensis* than in *C. hongkongensis*. Of these, glycine, alanine, glutamic acid, proline and serine were the predominant amino acids, consistent with the previous studies [14,15]. Glycine and alanine are both responsible for the sweetness of seafood, mainly contributing to the unique sweet flavor and complex texture of seafood [14,16]. Glutamic acid is the primary source of umami agent in seafood [17]. Proline, as an important component of collagen, is closely related to the flexibility and gelatinous texture of food [18]. Serine is the active center of many digestive enzymes that is involved in the metabolism and transformation of proteins in seafood, and has an impact on the freshness of seafood [19]. The amino acid composition in oysters is known to vary with genetic background, rearing environment, and seasonal conditions [20,21,22]. In this study, the differences in amino acid compositions between *C. hongkongensis* and *C. ariakensis* were likely attributable to species differences that jointly influenced the oyster physiology and biochemical profile. *C. ariakensis* exhibited higher total hydrolyzed amino acid contents, including several flavor-related amino acids such as glycine, alanine, and glutamic acid, which are known to contribute to the sweet and umami taste of seafood. These differences could influence consumer preference and the suitability of each species for different culinary applications, such as raw consumption, cooking, or processing. Moreover, the differential expression of proteins involved in protein turnover and extracellular matrix organization may affect the texture, water-holding capacity, and shelf-life stability of oyster meat. Therefore, our findings provide molecular-level insights that could inform quality evaluation and product development in the oyster industry. However, it should be noted that the present study measured total hydrolyzed amino acids, which include both protein-bound and free amino acids. Future studies specifically quantifying the free amino acid pool will be needed to directly link amino acid composition to sensory properties. Meanwhile, we acknowledge that environmental and seasonal factors may influence amino acid composition, and that our common-garden sampling design minimizes but does not completely exclude such effects.

### 3.2. Proteomic Analysis in C. hongkongensis and C. ariakensis

**Figure 2:**
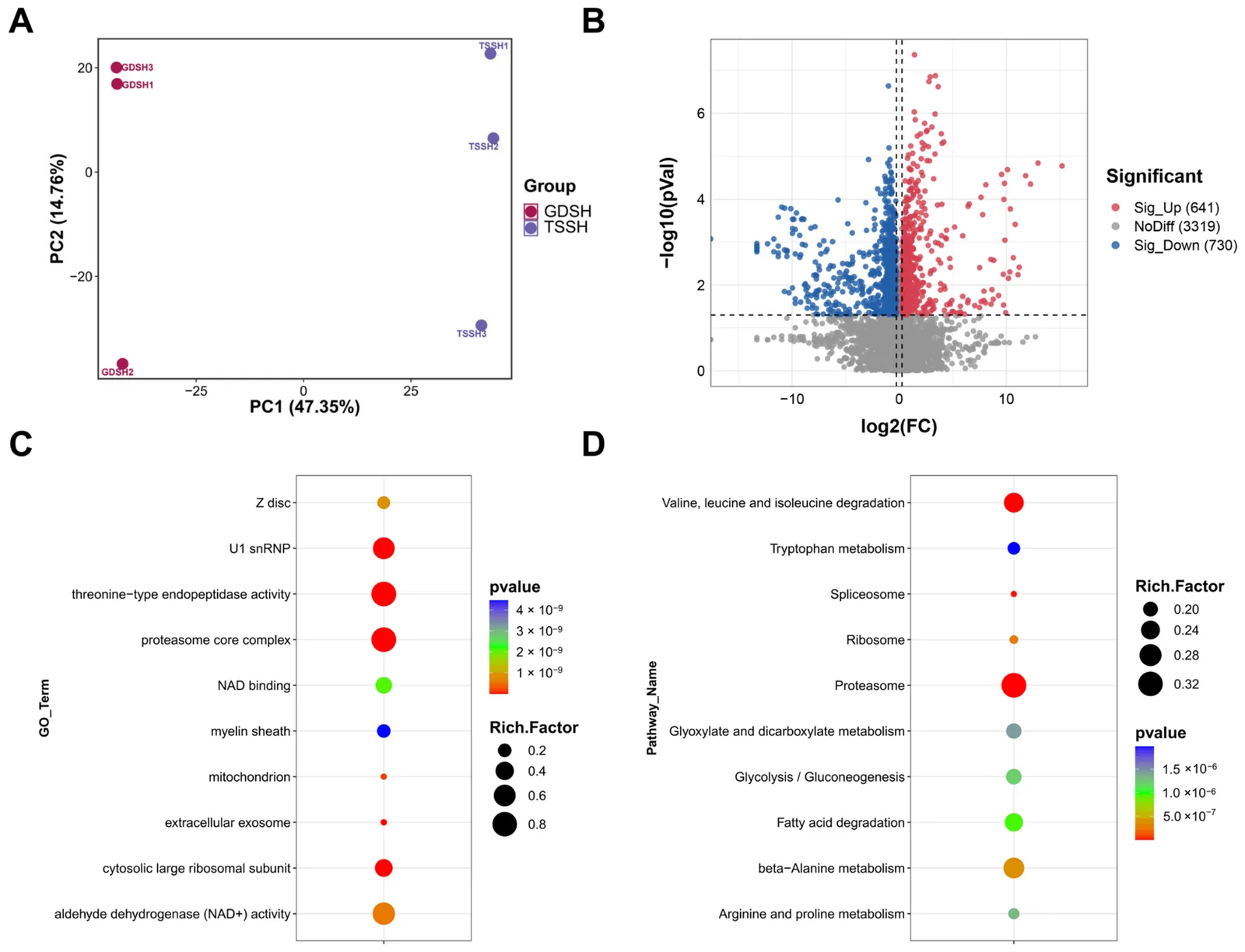
Proteomic analysis comparing *C. hongkongensis* and *C. ariakensis* (*n* = 3). (**A**) PCA analysis of proteins. (**B**) Volcano diagram of DEPs. (**C**) The top 10 significant GO enrichment pathways of DEPs. (**D**) The top 10 significant KEGG enrichment pathways of DEPs.

Proteomics was employed to explore the differences in protein expression between *C. hongkongensis* and *C. ariakensis*. It should also be noted that protein identification relied on the *C. gigas* reference proteome, and sequence divergence among *Crassostrea* species may affect the depth of proteome coverage. A total of 4871 proteins and 23,728 peptides were qualified. The PCA plot visually reflected the trend of differences between groups. In this study, PCA results showed that the samples of *C. hongkongensis* and *C. ariakensis* were clearly separated, with PC1 explaining 47.35% of the variance (Figure 2A). A volcano plot analysis showed that a total of 641 and 730 proteins were significantly upregulated and downregulated, respectively, in the comparison of *C. hongkongensis* and *C. ariakensis* (Figure 2B). The top nine DEPs with the smallest *p*-values are shown in a box plot, demonstrating that eight DEPs—A0A8B8A4U1, A0A8B8CTC8, A0A8B8DQA7, A0A8B8B2I0, A0A8B8CDJ4, A0A8B8E148, A0A8B8CFS6, and A0A8B8DIE6—were significantly upregulated in *C. hongkongensis*, and only the A0A8B8DT25 protein was significantly upregulated in *C. ariakensis* (Figure S1).

GO enrichment analysis indicated that the DEPs were significantly enriched in the molecular process of threonine-type endopeptidase activity and the cellular processes of proteasome core complex, U1 snRNP, and cytosolic large ribosomal subunit (Figure 2C and Figure S2). The proteasome core particle (20S proteasome) is a cylindrical macromolecular complex that constitutes the catalytic core of the 26S proteasome and mediates the ATP-independent proteolysis of ubiquitinated and misfolded proteins [23]. The cytosolic large ribosomal subunit is an essential component of eukaryotic ribosomes and serves as the catalytic center for peptide bond formation, thereby directly executing the elongation phase of protein synthesis [24]. U1 snRNP is a fundamental component of the spliceosome, responsible for recognizing the 5′ splice site and catalyzing the initial step of pre-mRNA splicing, thereby facilitating the generation of mature mRNA [25]. Generally, *C. hongkongensis* grows faster and has stronger adaptability to high-salt environments, and the enrichment of the ribosomal subunit pathway may be conducive to faster tissue growth and metabolism [26,27]. *C. ariakensis* is better adapted to low-salt estuarine environment and grows relatively slowly, and it may have a lower rate of protein synthesis but higher protein stability. Similarly, the enrichment results of DEP domains using the InterPro database were consistent with those of GO enrichment (Figure S3). Furthermore, the KEGG enrichment results showed the DEPs were markedly involved in the pathways of proteasome, valine, leucine and isoleucine degradation, spliceosome, ribosome, and other amino acid metabolism-related pathways (Figure 2D and Figure S4). The degradation of proteins by proteasomes can release free amino acids and short peptides, which may affect the composition and content of free amino acids in tissues, thereby influencing flavor (sourness, sweetness) [28]. This may be related to the differences in flavor amino acids such as glycine and alanine between *C. hongkongensis* and *C. ariakensis*. Overall, these pathways were involved in the core processes of protein metabolism, ranging from protein synthesis to post-transcriptional regulation and finally to protein degradation. These results indicated that there were significant differences between *C. hongkongensis* and *C. ariakensis* in terms of amino acid composition and protein expression profiles, which may be related to growth characteristics and environmental adaptability.

### 3.3. Sequencing and De Novo Assembly of Transcriptome

**Figure 3:**
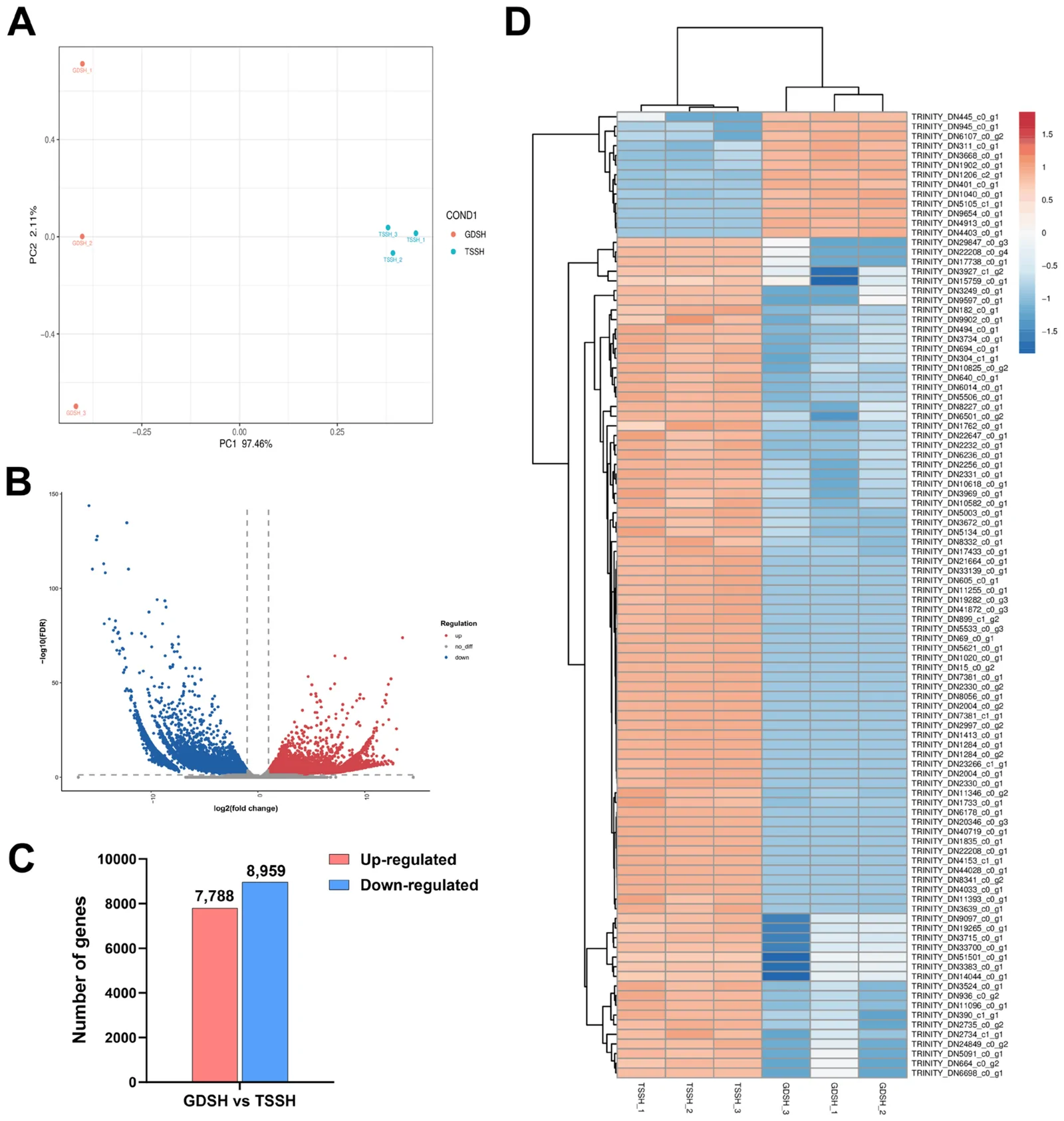
Transcriptomic analysis comparing *C. hongkongensis* and *C. ariakensis* (*n* = 3). (**A**) PCA plots of unigenes. (**B**) Volcano plot of DEGs. The red dots correspond to upregulated genes (with fold change > 2), the green dots correspond to downregulated genes (with fold change < 0.5), and the gray dots represent genes with insignificant differential expression. (**C**) Statistics of the upregulated and downregulated DEGs. (**D**) Hierarchical cluster heatmap of DEGs.

To further investigate the molecular mechanism of protein metabolism, de novo assembly of the transcriptome was employed between *C. hongkongensis* and *C. ariakensis*. After filtering of the raw reads, 5.64 Gb of clean bases were obtained for *C. hongkongensis* and 5.57 Gb for *C. ariakensis* (Table 1). For the *C. hongkongensis* samples, the mean Q20 and Q30 values were 99.37% and 96.95%, respectively, and the GC percentage was 40.74%. For the *C. ariakensis* samples, Q20, Q30%, and GC content were 99.29%, 96.62%, and 38.39%, respectively. A total of 65,542 unigenes were obtained after *de novo* assembly and annotated using the NR, GO, KEGG, Pfam, SwissProt, and eggNOG databases to understand their functions (Table 2). Subsequently, PCA analysis exhibited significant differences between samples in the two groups, with 97.46% explanatory power (PC1) (Figure 3A). Differential expression of unigenes was screened with the criteria of FDR < 0.05 and FC > 2. A total of 16,747 DEGs were identified, including 7788 upregulated and 8959 downregulated unigenes (Figure 3B,C). The large number of DEGs indicated obvious differences between *C. hongkongensis* and *C. ariakensis*. The cluster heatmap of the top 100 DEGs with the smallest FDR values showed that most unigenes were upregulated in *C. hongkongensis* and downregulated in *C. ariakensis* (Figure 3D).

### 3.4. GO Terms and KEGG Enrichment Analysis of DEGs

**Figure 4:**
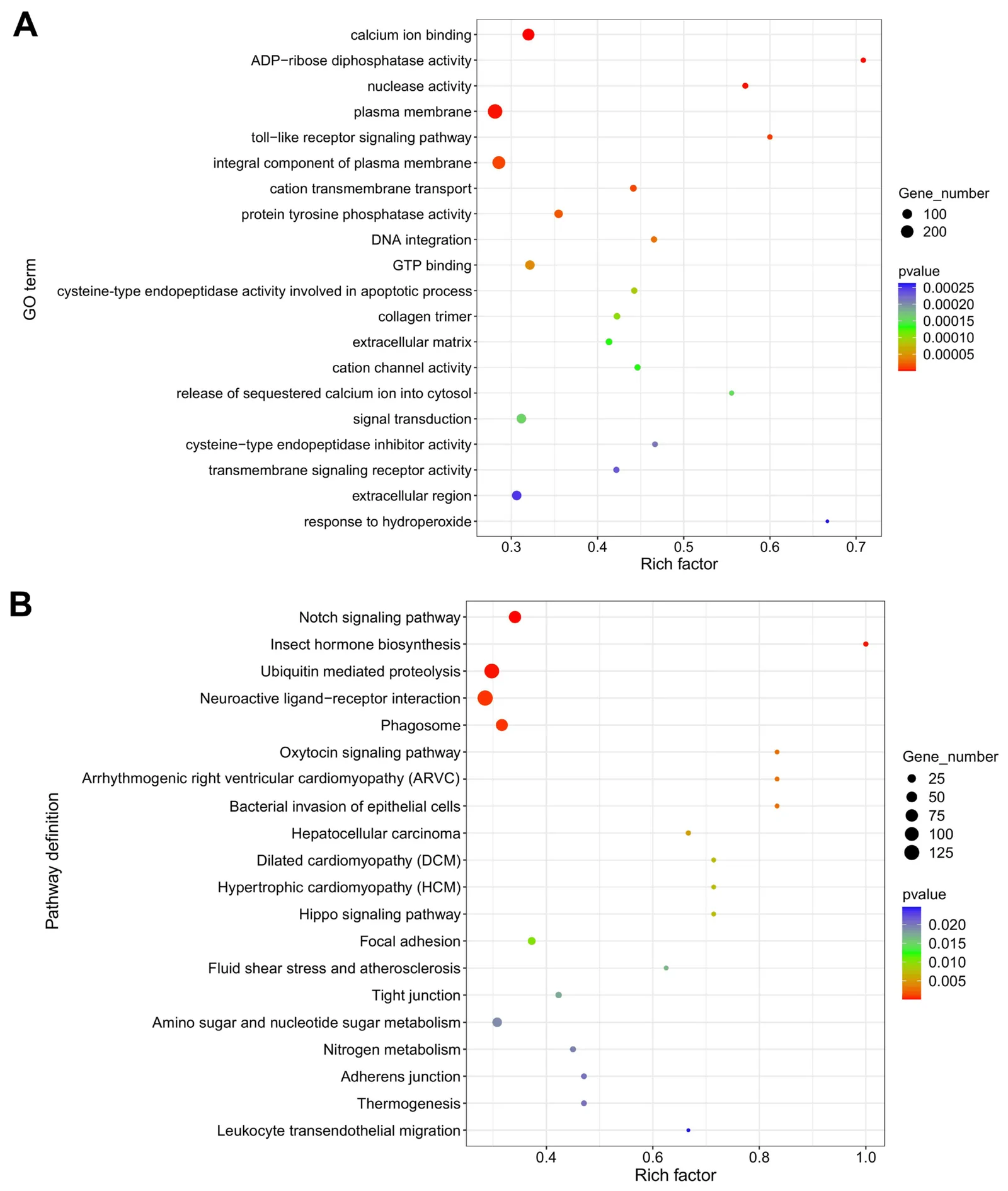
GO terms and KEGG enrichment analysis of DEGs between *C. hongkongensis* and *C. ariakensis*. (**A**) The top 20 significant GO terms and (**B**) the top 20 KEGG pathways of DEG enrichment.

GO terms and KEGG enrichment analysis of DEGs were conducted to further elucidate the differences in protein metabolism between *C. hongkongensis* and *C. ariakensis*. GO enrichment analysis was divided into three categories including molecular function, cellular component, and biological process. DEGs were significantly enriched in molecular functions related to calcium ion binding (GO:0005509), ADP-ribose diphosphatase activity (GO:0047631), nuclease activity (GO:0004518) and protein tyrosine phosphatase activity (GO:0004725), the cellular component of plasma membrane (GO:0005886) and integral component of plasma membrane (GO:0005887), and the biological processes of toll-like receptor signaling pathway (GO:0002224), cation transmembrane transport (GO:0098655), DNA integration (GO:0015074), and so on (Figure 4A). These GO terms were related to various biological processes such as signal transduction, immunity, protein degradation, ion transport, nucleic acid metabolism. Most of these processes took place in the plasma membrane and depended on calcium ion signaling or protein phosphorylation/dephosphorylation. Nuclease activity and DNA integration are associated with genomic defense or transposon activity, while ADP-ribosylation metabolism is involved in DNA repair or signal regulation [29,30]. The differences in ADP-ribosylation metabolism and DNA integration processes may suggest that the two species have differences in dealing with DNA damage or the activities of genetic elements. This might be a genetic characteristic formed as a result of their long-term adaptation to different environments (low salinity vs. high salinity). Additionally, the toll-like receptors are a crucial type of pattern recognition receptors (PRR) in the innate immune system, which can recognize and trigger immune responses. Bivalves rely on innate immunity to defend against the invasion of various bacteria, viruses and other microorganisms [31]. In invertebrates such as oysters, the Toll-like receptor signaling pathway plays a crucial role in innate immune recognition and inflammation regulation [32]. It has been reported that the Toll-like receptor signaling pathway was significantly enriched in *C. hongkongensis* infected with *V. harveyi* [33]. Similarly, under the infection of other pathogenic bacteria such as *V. splendidus*, *Edwardsiella tarda* and *V. parahaemolyticus*, the results indicated that toll receptors were significantly enriched in oysters, playing an important role in the regulation of pathogen proliferation and inflammatory immune response [34,35,36]. In this study, our results were consistent with previous studies, suggesting that the active immune response and protein metabolism in *C. hongkongensis* and *C. ariakensis* may be related to environmental adaptability.

Furthermore, all DEGs were subjected to KEGG enrichment analysis to predict the pathways. The results indicated that DEGs were significantly enriched in the Notch signaling pathway, insect hormone biosynthesis, ubiquitin mediated proteolysis, neuroactive ligand-receptor interaction, phagosome, and so on (Figure 4B). Consistently, ubiquitination can regulate protein degradation, transport and signal transduction, and is crucial for protein homeostasis, DNA repair, cell cycle regulation, and immune responses [37]. Phagocytosis is a fundamental mechanism in innate immune defense and serves as a bridge between innate immunity and adaptive immunity [38]. The significant enrichment of ubiquitination and phagosome may imply differences between the two species in their ability to recognize and eliminate pathogens, as well as protein degradation and renewal. Notably, the Notch signaling pathway plays a vital role in regulating the activation and infiltration of various immune cells (e.g., macrophages and T cells), cell proliferation and skeletal muscle development [39,40]. The exoskeleton of bivalves contains resistant components and matrix proteins, contributing to defense against pathogen invasion, repairing the shell, and energy metabolism for movement [41,42]. In Lu’s study, proteomics and transcriptomics were used to analyze the stress response of pearl oysters to nuclear transplantation; the results also significantly enriched the same pathways such as neuroactive ligand-receptor interaction, Toll-like receptor signaling pathway, and Notch signaling pathway [43]. This highlighted the significance of these pathways in the immune system and the adaptation mechanism of oysters. In this study, the cross-talk between Notch and innate immune pathways (e.g., Toll-like receptor signaling, phagosome) may suggest potential differences in immune readiness between the two species; however, direct functional assays are required to confirm this interpretation. These molecular differences may contribute to, but do not directly demonstrate, differences in muscle function or pathogen resistance.

### 3.5. Correlation Analysis Between Transcriptomics and Proteomics

**Figure 5:**
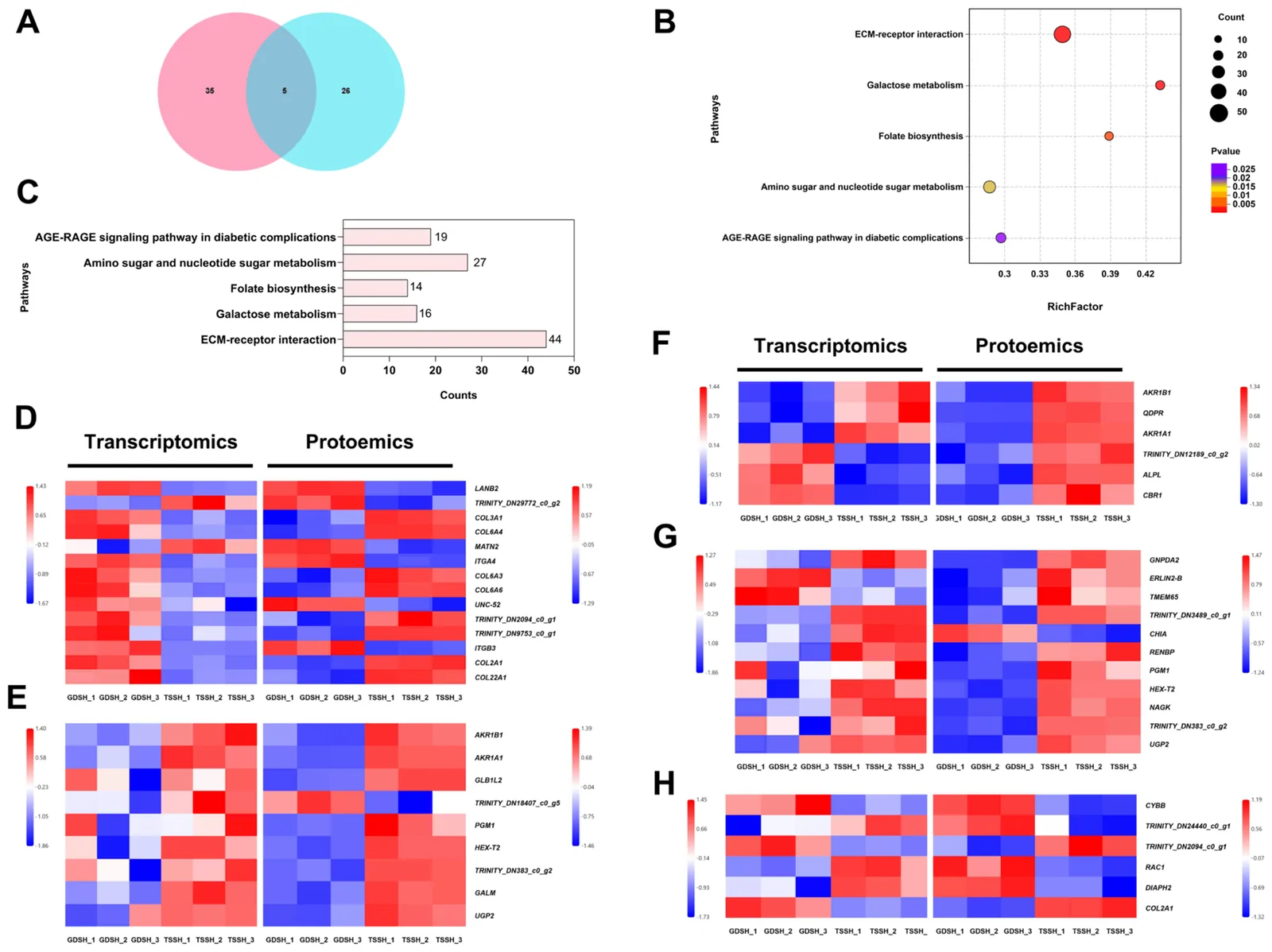
Correlation analysis between transcriptomics and proteomics. (**A**) Venn diagram of the significant KEGG pathways of the proteomics and transcriptomics. (**B**) Scatter plot for the five significant KEGG pathways shared by the proteomics and transcriptomics. (**C**) The numbers of DEGs in each of the five common KEGG pathways. The heatmap showing the expression patterns of the DEGs/DEPs in the pathway of (**D**) ECM–receptor interaction, (**E**) galactose metabolism, (**F**) folate biosynthesis, (**G**) amino sugar and nucleotide sugar metabolism, and (**H**) the AGE-RAGE signaling pathway in diabetic complications.

We conducted a Venn analysis of the significantly enriched KEGG pathways from proteomics and transcriptomics to identify common pathways. The Venn diagram showed that, out of 40 proteomic and 31 transcriptomic pathways, five pathways were shared between the two omics layers (Figure 5A). These five common pathways included ECM–receptor interaction, galactose metabolism, folate biosynthesis, amino sugar and nucleotide sugar metabolism, and the AGE-RAGE signaling pathway in diabetic complications. These pathways represented robust and concordant molecular differences between the two species, as they were consistently detected at both the transcriptional and protein levels (Figure 5B). In particular, the shared enrichment of the ECM–receptor interaction pathway was the most significant pathway and included 44 DEGs, indicating differences in cell–matrix adhesion and tissue architecture between *C. hongkongensis* and *C. ariakensis* (Figure 5C). We further analyzed the transcriptional expression levels of the DEGs and the protein expression levels of the corresponding DEPs across each of the five common pathways (Figure 5D–H). Interestingly, we observed that the expression trends of several DEGs and their corresponding DEPs were opposite. For example, in the ECM–receptor interaction pathway, transcript levels of DEGs such as *COL3A1*, *COL2A1*, *COL22A1*, and *COL6A4* were significantly upregulated in *C. hongkongensis* compared to *C. ariakensis*, whereas their protein expression levels showed significantly lower abundance in *C. hongkongensis* (Figure 5D). These inconsistencies suggest that these DEGs may be subject to post-transcriptional or post-translational regulation, including miRNA-mediated translational repression, altered protein stability, or enhanced proteasomal degradation, resulting in inconsistent mRNA and protein levels. Given that *C. hongkongensis* exhibits higher proteasome activity, it is plausible that these collagen proteins are rapidly degraded despite elevated transcript levels, contributing to the species-specific extracellular matrix remodeling strategy. However, direct evidence for such regulation was not obtained in this study. These observations are correlative, and functional validation would be required to establish causality.

### 3.6. Gene Set Enrichment Analysis (GSEA) Based on the Common KEGG Enrichment Pathways of Proteomics and Transcriptomics

**Figure 6:**
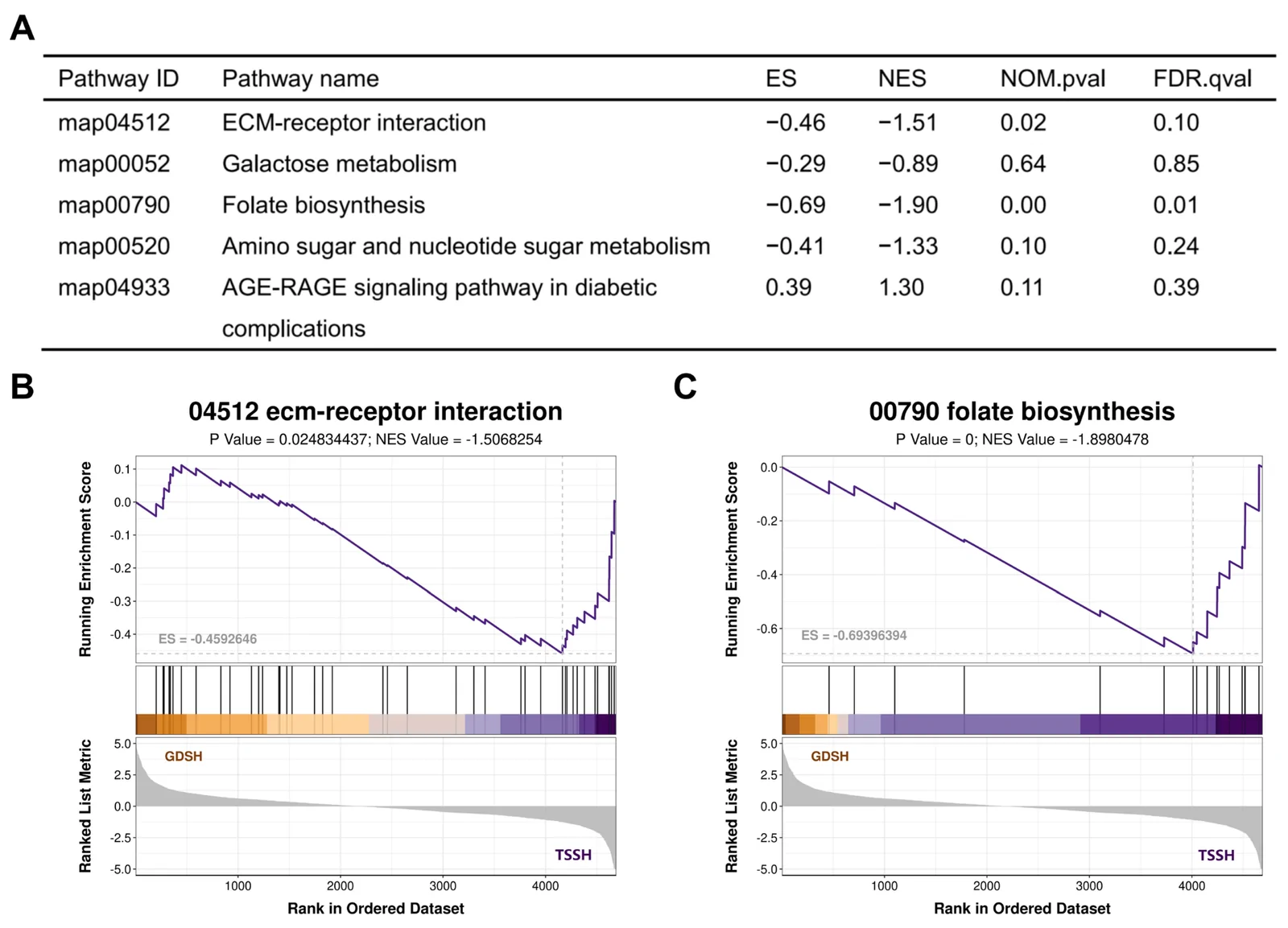
GSEA enrichment scores (normalized enrichment score, NES) for the five KEGG pathways shared between transcriptomics and proteomics. (**A**) The five KEGG-enrichment plots of the proteomic results. NES, normalized enrichment scores. (**B**,**C**) KEGG enrichment ES line chart of the pathways of the ECM–receptor interaction and the folate biosynthesis, respectively.

To further assess the directional changes of the five shared KEGG pathways, we performed GSEA using the full proteomic datasets. GSEA showed negative enrichment of the ECM–receptor interaction, galactose metabolism, folate biosynthesis, and amino sugar and nucleotide sugar metabolism pathways in *C. hongkongensis* relative to *C. ariakensis*. It observed that the pathways of ECM–receptor interaction, galactose metabolism, folate biosynthesis, and amino sugar and nucleotide sugar metabolism showed negative enrichment scores (ES < 0) in *C. hongkongensis* compared to *C. ariakensis*, while the AGE-RAGE signaling pathway in diabetic complications exhibited a positive but non-significant enrichment trend (ES = +0.39, *p* > 0.05) (Figure 6A). Remarkably, ECM–receptor interaction (ES = −0.46, |NES| = 1.51) (NOM *p*-value < 0.05) and folate biosynthesis (ES = −0.69, |NES| = 1.90) (FDR = 0.005) were statistically significant (Figure 6B,C), suggesting their potential involvement in shaping amino acid composition and protein expression profiles. Amino sugar and nucleotide sugar metabolism is directly related to the synthesis of UDP-N-acetylglucosamine (UDP-GlcNAc), a necessary precursor for chitin and glycoproteins [44]. Galactose metabolism is associated with amino sugar metabolism through uridine diphosphate glucose isomerization [45]. ECM components (e.g., collagen, laminin, fibronectin) and their receptors play critical roles in shell formation, muscle attachment, and immune responses [46,47]. Folate (vitamin B9) is a cofactor in the single-carbon metabolism process and is crucial for nucleotide synthesis, DNA methylation, and amino acid homeostasis [48,49]. These common metabolic pathways may imply differences in chitin-based matrix synthesis ability, protein homeostasis, or carbohydrate metabolism between *C. hongkongensis* and *C. ariakensis*. Additionally, the AGE-RAGE signaling pathway is involved in oxidative stress, inflammation, and metabolic dysregulation [50,51]. The shared enrichment of this pathway may be associated with differences in stress resilience or inflammatory response. Notably, many pathways that existed solely in the proteomic data (such as the Notch signaling pathway and proteasome) may be mainly controlled at the post-transcriptional level rather than at the transcriptional level. This suggests potential differences between the two species at the transcriptional and proteomic levels under conditions of rapid environmental adaptation. Collectively, these five common pathways were involved in extracellular matrix/carbohydrate metabolism and stress signaling, consistent with our previous results on protein homeostasis and muscle function. These molecular differences may contribute to the observed variation in nutritional traits, although direct phenotypic measurements were not performed in this study.

## 4. Conclusions

In this study, we conducted a comparative analysis of the amino acid composition and protein expression profiles between *C. hongkongensis* and *C. ariakensis* using transcriptomics and proteomics. Our results showed that total amino acid content and the concentrations of individual amino acids were higher in *C. ariakensis* compared to *C. hongkongensis*. Among them, glycine, alanine, glutamic acid, proline and serine have particularly abundant contents. The integration of proteomics (1371 DEPs) and transcriptomics (16,747 DEGs) jointly identified five common KEGG pathways: ECM–receptor interaction, galactose metabolism, folate biosynthesis, amino sugar and nucleotide sugar metabolism, and the AGE-RAGE signaling pathway in diabetic complications. Among these, GSEA further indicated that ECM–receptor interaction and folate biosynthesis were significantly downregulated in *C. hongkongensis* compared to *C. ariakensis*. Overall, these findings provide a clearer understanding of the molecular basis underlying species-specific differences in amino acid composition and protein expression profiles in oysters, and offer a foundation for the further investigation of nutritional differences and their potential implications for oyster aquaculture.

## Figures and Tables

**Table 1:** Summary of the RNA-seq analyses.

Sample	Raw Reads	Raw Bases	Clean Reads	Valid Bases	Valid%	Q20%	Q30%	GC%
GDSH_1	36,859,576	5.53G	36,363,642	5.37G	98.65	99.33	96.94	41.10
GDSH_2	39,623,492	5.94G	39,077,998	5.77G	98.62	99.32	96.82	40.37
GDSH_3	39,526,530	5.93G	39,067,474	5.78G	98.84	99.47	97.08	40.74
TSSH_1	35,510,940	5.33G	34,715,210	5.10G	97.76	99.26	96.50	38.01
TSSH_2	3,9443,544	5.92G	38,662,494	5.69G	98.02	99.29	96.66	38.62
TSSH_3	41,091,592	6.16G	40,291,164	5.93G	98.05	99.32	96.71	38.54

Notes: Clean reads: The number of reads after removing low-quality sequences. The subsequent analysis is based on clean reads. Error rate: Base error rate. Q20 and Q30, the percentage of bases with Phred values > 20 and >30, respectively. GC content: the GC ratio of the total base number. Q20 > 95%, Q30 > 90%, and GC content of 40–50% are generally considered indicative of high-quality RNA-seq data. In this study, Q20 and Q30 exceeded 99% and 96%, respectively, and GC content was within the expected range, confirming the high quality of the sequencing data.

**Table 2:** The summary of unigene annotation using different databases.

Databases	Number	Ratio (%)
Total Unigenes	65,542	100.00
GO	13,905	21.22
KEGG	6422	9.80
Pfam	20,236	30.87
SwissProt	12,683	19.35
eggNOG	16,213	24.74
NR	9252	14.12
TF	635	0.97

## Data Availability

The original contributions presented in this study are included in the article/Supplementary Material. Further inquiries can be directed to the corresponding authors.

## Supplementary Materials

The following supporting information can be downloaded at: https://www.mdpi.com/article/10.3390/foods15203606/s1, Figure S1. Proteomic analysis between *C. hongkongensis* and *C. ariakensis*. Figure S2. The top 20 significant GO terms of DEPs between *C. hongkongensis* and *C. ariakensis*. Figure S3. The top 20 significant KEGG pathways of DEPs between *C. hongkongensis* and *C. ariakensis*. Figure S4. The top 10 significant KEGG pathways of DEPs between *C. hongkongensis* and *C. ariakensis* by InterPro database.
