# Supplementary material for: Integrated Transcriptomics and Proteomics Reveal Amino Acid Composition and Protein Expression Profiles of Crassostrea hongkongensis and Crassostrea ariakensis
Source: Foods. 2026 Oct 10;15(20):3606. doi: 10.3390/foods15203606 (PMC13704749; doi:10.3390/foods15203606)

Supplementary materials

Figure S1. Proteomic analysis between *C. hongkongensis* and *C. ariakensis*. (A) The top 9 DEPs with the smallest P-value in the comparison of *C. hongkongensis* and *C. ariakensis*. (B) Classification of GO enrichment and (C) KEGG enrichment pathways of DEPs. GDSH: *C. hongkongensis,* TSSH: *C. ariakensis*.

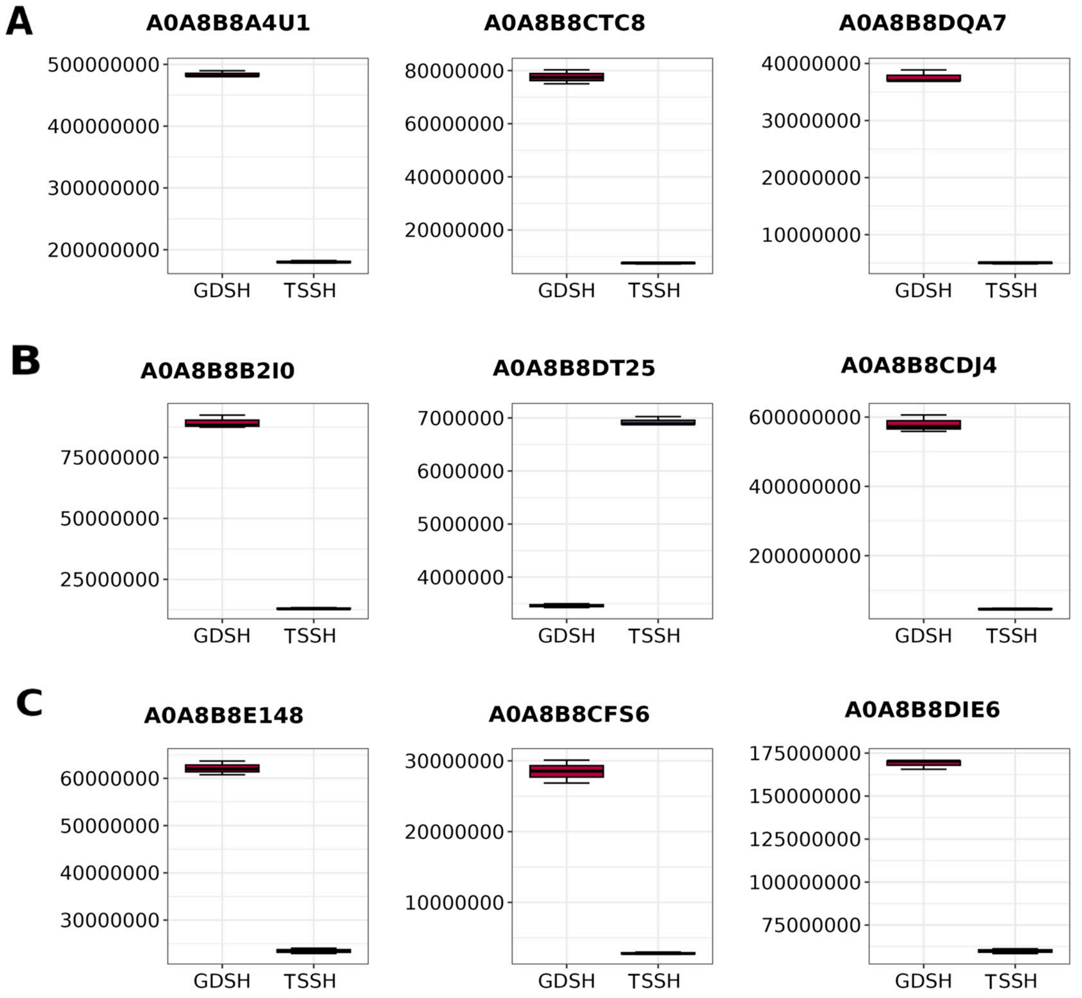

Figure S2. The top 20 significant GO terms of DEPs between *C. hongkongensis* and *C. ariakensis*.

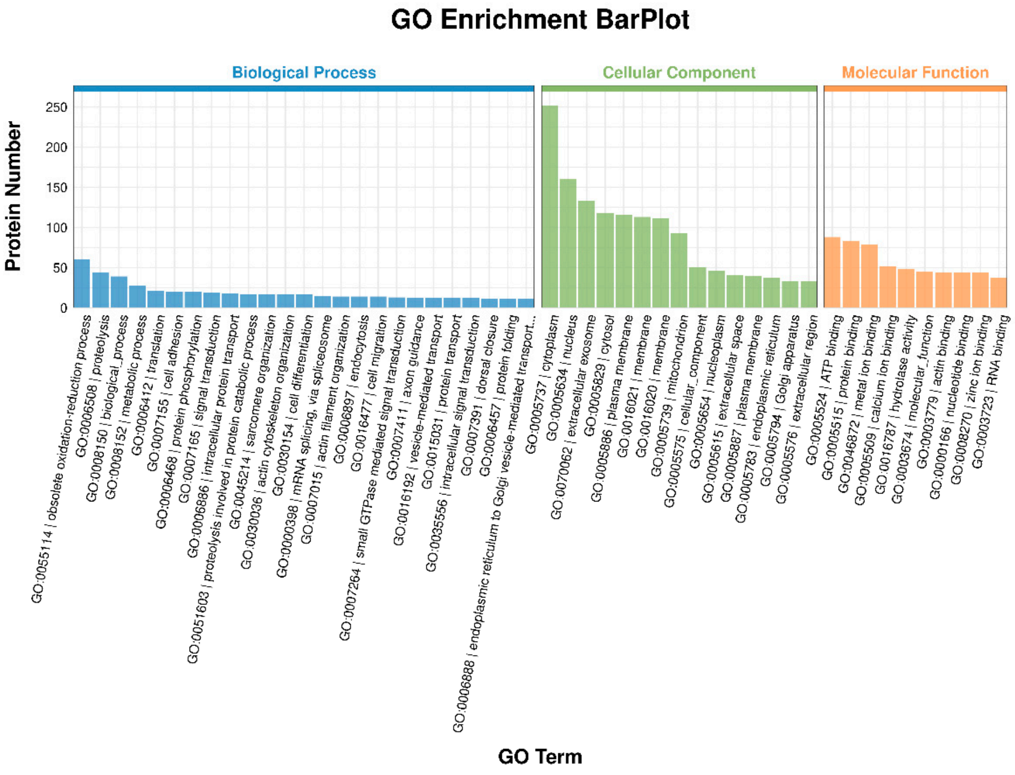

Figure S3. The top 20 significant KEGG pathways of DEPs between *C. hongkongensis* and *C. ariakensis*.

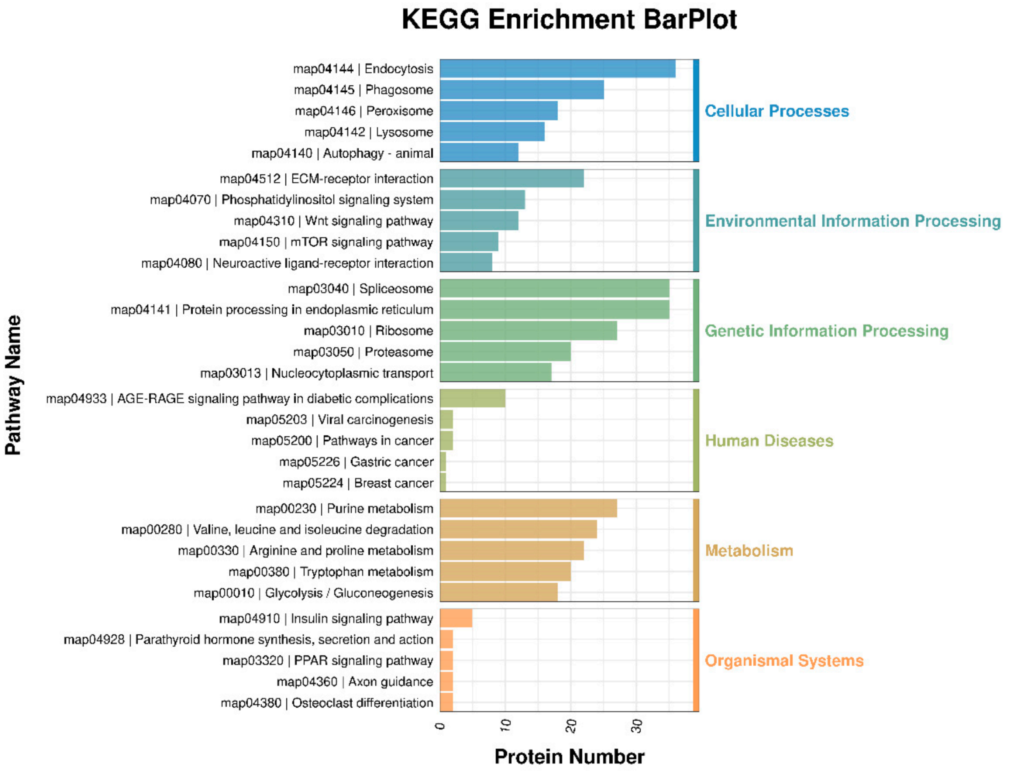

Figure S4. The top 10 significant KEGG pathways of DEPs between *C. hongkongensis* and *C. ariakensis* by InterPro database.

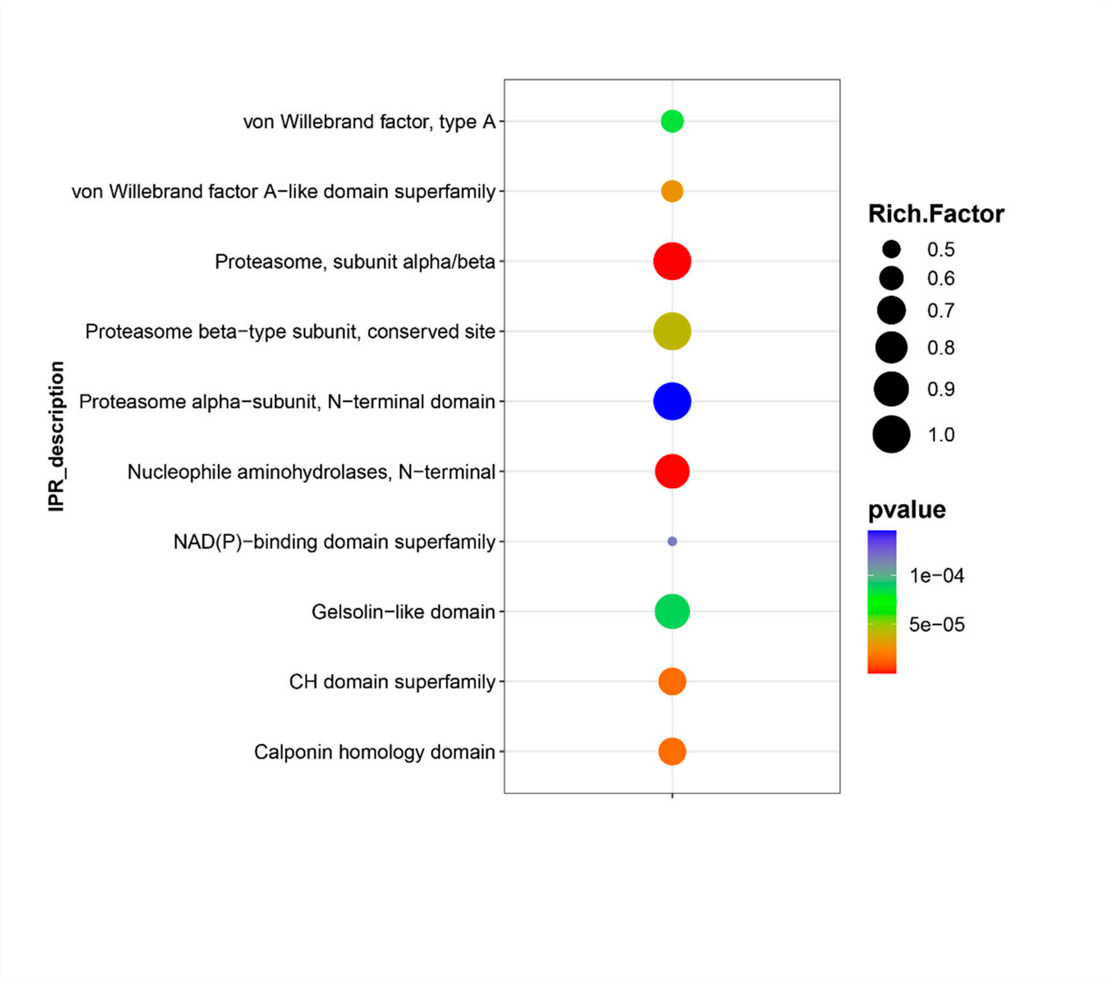

Supplement: Supplementary file 1 [file foods-15-03606-s001.zip › foods-4558050-supplementary.pdf]
